# Intra-Abdominal Manifestations of von Recklinghausen's Neurofibromatosis

**DOI:** 10.4103/1319-3767.39623

**Published:** 2008-04

**Authors:** Rajul Rastogi

**Affiliations:** Yash Diagnostic Center, Yash Hospital and Research Center, Civil Lines, Kanth Road, Moradabad, India

**Keywords:** von Recklinghausen, neurofibroma, gastric outlet obstruction

## Abstract

Neurofibromatosis type-1 (NF1), also known as von Recklinghausen disease, is a common autosomal dominant condition with an approximate incidence of one per 3000 births. NF1 involves multiple systems of the body. Abdominal involvement occurs in the form of neurofibroma and tumour growth in the liver, mesentery, retroperitoneum, gastric and bowel. Gastrointestinal neoplasms have a reported occurrence of 2–25%. Two cases are reported herein as well as a review of the literature of the intra-abdominal manifestations of NF1, including a discussion on the radiological appearance and diagnosis. The article provides an insight into the intriguing variety of clinical problems that such patients may present.

Neurofibromatosis (NF) is an autosomal dominant disorder that can involve any organ system. NF is not a single entity but a group of heterogeneous multisystemic neurocutaneous disorders. There are two distinct types: neurofibromatosis type-1 (NF1) or von Recklinghausen disease, which affects 85% of patients, and neurofibromatosis type-2 (NF2) or bilateral acoustic neuromas/schwannomas, which affects 10% of patients. Abdominal involvement in patients with NF1 has been described in the form of neurofibromas within the liver, mesentery, retroperitoneum and gastrointestinal (GI) tract. Large-bowel intussusception has also been reported. Small-bowel leiomyomas, adenocarcinomas with neuroendocrine function, GI tract vasculopathy, GI tract bleeding, pseudo-obstruction and protein-losing enteropathy also may occur.

## CASE REPORTS

Two patients of NF1 with different sets of complaints were referred to our department for computed tomography (CT) scan of the abdomen.

### Case 1

A 35-year-old male patient with a 5–6-month history of vague, intermittent abdominal pain presented to us for CT examination of the abdomen. An ultrasound of the abdomen revealed lymphadenopathy at the porta hepatis, peripancreatic and retroperitoneal regions.

**Figure 1:**
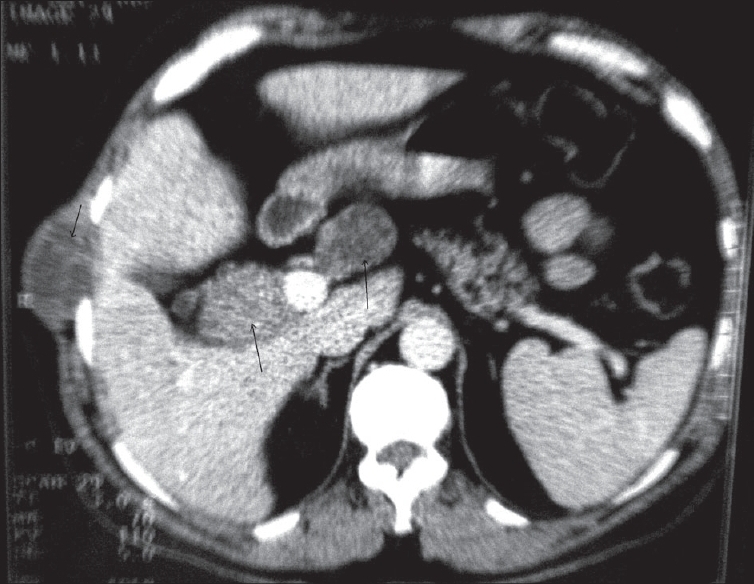
Contrast-enhanced axial CT image showing neurofibromas at the porta hepatis, peripancreatic region and right lateral abdominal wall

**Figure 2:**
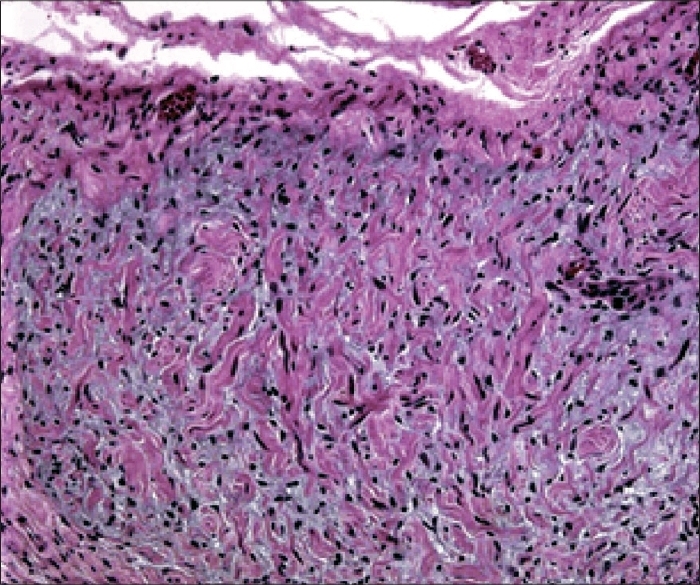
Photomicrograph (H and E stain) of an abdominal neurofibroma shows densely arranged spindle cells with curved nuclei mixed with eosinophilic collagen and basophilic myxoid matrix

Contrast-enhanced CT examination was performed through the abdomen, from the domes of the diaphragm to the pubic symphysis. The examination revealed multiple, well-defined, oval to round, soft tissue attenuating masses in the liver, porta hepatis, peripancreatic region and retroperitoneum. In addition, a similar lesion was noted in the right lateral abdominal wall [Figure 1]. The lesions were hypodense on non-contrast images with minimal to mild heterogeneous enhancement on post-contrast images. No other abnormality was noted. Radiological probability of neurofibromas was suggested, which was confirmed by histopathological diagnosis [Figure 2].

This patient was managed conservatively on medical therapy and followed up for 3–4 months, during which the patient did well. Patient was suggested to follow up at six-monthly intervals.

### Case 2

A 43-year-old male patient came for CT examination of the abdomen with complaints of recurrent vomiting and occasional hemetemesis, and abdominal distension especially after meals. An ultrasound of the abdomen was unremarkable.

**Figure 3:**
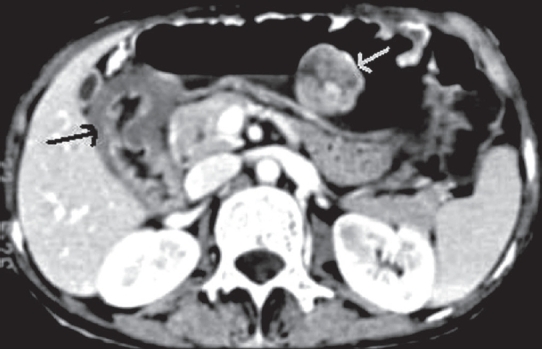
Contrast-enhanced axial CT image showing a neurofibroma along the greater curvature of the stomach along with diffuse thickening of the gastric outlet due to diffuse neurofibromatosis

Contrast-enhanced CT examination was performed through the abdomen, from the domes of the diaphragm to the pubic symphysis. The examination revealed a large, well-defined, polypoidal soft tissue mass with mild to moderate post-contrast enhancement along the greater curvature of the stomach. In addition, diffuse thickening of gastric wall in the region of the pyloric antrum was observed with over-distension of the stomach suggesting gastric outlet obstruction [Figure 3]. Post-contrast enhancement of the thickened wall was insignificant. No associated lymphadenopathy was noted. As the patient was a case of NF1, the radiological possibility of gastric neurofibroma with gastric outlet obstruction possibly due to submucosal neurofibroma was suggested. However, the possibility of gastric adenocarcinoma was not ruled out. Endoscopic biopsy and tissue diagnosis confirmed the polypoidal mass to be a neurofibroma, and the thickened wall to be submucosal neurofibromatosis.

The patient underwent partial gastrectomy along with Billroth type-II gastrojejunostomy. The post-operative as well as the 6-month follow-up period was uneventful.

## DISCUSSION

NF1 is a multisystemic disorder that may affect any organ in the body. Clinical presentation depends on the organ system involved. In NF1, gastrointestinal tumors occur most frequently followed by thoracic tumors, arterial involvement and endocrine tumors in the decreasing order of their frequency.[1,2] Abdominopelvic involvement in NF1 is primarily extraperitoneal. Although lesions are mostly detected in the abdominopelvic wall and lumbosacral plexus, retroperitoneal and pelvic involvement is more common and usually affects important organs.[3] GI tract involvement includes neurofibromas within the liver, mesentery, retroperitoneum, stomach, small and large bowel, rectum, small-bowel leiomyomas, small-bowel adenocarcinoma with neuroendocrine function, GI tract vasculopathy and bleeding, pseudo-obstruction and protein-losing enteropathy. Patients may present with abdominal pain, nausea, abdominal distension, diarrhea, constipation, bowel perforation or GI tract bleeding.

In one study, majority of the neoplasms were located in the small intestine (72%). Neurofibromas, found in 52% of patients, were the most frequently diagnosed benign neoplasms followed by leiomyomas (13%), ganglioneurofibromas (9.8%) and gastrointestinal stromal tumor (6.5%). Adenocarcinoma was present in 23% of patients. Neurofibromas may be diffuse and submucosal, making radiographic visualization sometimes very difficult. Auerbach plexus is the usual site of origin. Benign neurofibromas undergo malignant transformation in 5–15%, especially in patients above the age of 40 years.[4]

Imaging plays an important role in the diagnosis, evaluation and follow-up of patients with abdominal manifestations of NF1. Barium studies may demonstrate intraluminal mass lesions. Sometimes, intussusception of the bowel may be detected along with intestinal obstruction. Features of malabsorption may also be seen.

Solitary neurofibroma is mostly hyperechoic with coarse internal echoes and is lobulated but with smooth and well-defined margins. Biliary obstruction may be demonstrated, and tumour metastasis into the liver along the portal vein may be seen as infiltrative hypoechoic masses around the porta hepatis and intrahepatic portal branches. Duplex color and spectral Doppler ultrasonography reveal the vascular complications of NF1 such as aneurysms and stenoses.

Biliary strictures and bile duct intraluminal neurofibromas are rarely associated with NF, but if present, may be visualized by antegrade or retrograde cholangiographic imaging.

CT examination may demonstrate solid fusiform masses along the distribution of nerves, with central areas of low attenuation and occasional calcification. The masses usually are well defined and have homogeneous low attenuation, equal to or slightly more than water, but lower than muscles.[5] Asymmetry in the size or attenuation of the mass may suggest malignant transformation.[6] They may also show mild to moderate heterogeneous post-contrast enhancement.

Magnetic resonance imaging (MRI) is considered to be the modality of choice. In one series, MR imaging added information in the initial and follow-up clinical evaluation of these patients.[3]

Surgical excision is the treatment of choice for all symptomatic tumours occurring in patients with NF1. As the benign tumours occurring in these patients always carry some risk of malignant degeneration, surgical excision of such tumours is advocated.[7] But as the risk of malignant degeneration and developing malignancy even after surgery cannot be calculated preoperatively, surgical excision of benign asymptomatic tumours is still controversial.

In conclusion, patients with NF1 with GI symptoms are at risk for GI neoplasms from which they are likely to experience significant morbidity. Due to its rarity, a high degree of suspicion is needed for timely diagnosis. Delay in the diagnosis of GI NF is common. The average interval from onset of GI symptoms to the diagnosis and detection of GI neoplasms was 2.8 years in one series.[4] Diagnosis may be difficult due to non-specific symptoms as well as the predominantly small bowel location. Imaging studies not only help in evaluation and localizing the lesions, but also help in identifying concomitant lesions and in the follow-up assessments of these patients.

## References

[1] Wander JV, Das Gupta TK (1977). Neurofibromatosis. Curr Probl Surg.

[2] Nordback P, Halkic N, Boumghar M (2000). Intrathoracic tumors in von Recklinghausen's neurofibromatosis. Schweiz Med Wochenschr.

[3] Zacharia TT, Jaramillo D, Poussaint TY, Korf B (2005). MR imaging of abdominopelvic involvement in neurofibromatosis type 1: A review of 43 patients. Pediatr Radiol.

[4] Bakker JR, Haber MM, Garcia FU (2005). Gastrointestinal neurofibromatosis: An unusual cause of gastric outlet obstruction. Am Surg.

[5] Lee JK, Hiken JN, Semelka RC, Lee JK, Sagel SS, Stanley RJ (1998). Retroperitoneum. Computed body tomography.

[6] Bass JC, Korobkin M, Francis IR, Ellis JH, Cohan RH (1994). Retroperitoneal plexiform neurofibromas: CT findings. AJR Am J Roentgenol.

[7] Cosgrove JM, Fischer MG (1988). Gastrointestinal neurofibroma in a patient with von Recklinghausen's disease. Surgery.

